# Emotion perception improvement following high frequency transcranial random noise stimulation of the inferior frontal cortex

**DOI:** 10.1038/s41598-017-11578-2

**Published:** 2017-09-12

**Authors:** Tegan Penton, Laura Dixon, Lauren Jayne Evans, Michael J. Banissy

**Affiliations:** 1 MRC Social, Genetic and Developmental Psychiatry Centre, Institute of Psychiatry, Psychology and Neuroscience, King’s College London, Denmark Hill, London SE5 8AF UK; 2 Department of Psychology, Goldsmiths, University of London, New Cross, London SE14 6NW UK

## Abstract

Facial emotion perception plays a key role in interpersonal communication and is a precursor for a variety of socio-cognitive abilities. One brain region thought to support emotion perception is the inferior frontal cortex (IFC). The current study aimed to examine whether modulating neural activity in the IFC using high frequency transcranial random noise stimulation (tRNS) could enhance emotion perception abilities. In Experiment 1, participants received either tRNS to IFC or sham stimulation prior to completing facial emotion and identity perception tasks. Those receiving tRNS significantly outperformed those receiving sham stimulation on facial emotion, but not identity, perception tasks. In Experiment 2, we examined whether baseline performance interacted with the effects of stimulation. Participants completed a facial emotion and identity discrimination task prior to and following tRNS to either IFC or an active control region (area V5/MT). Baseline performance was a significant predictor of emotion discrimination performance change following tRNS to IFC. This effect was not observed for tRNS targeted at V5/MT or for identity discrimination. Overall, the findings implicate the IFC in emotion processing and demonstrate that tRNS may be a useful tool to modulate emotion perception when accounting for individual differences in factors such as baseline task performance.

## Introduction

Daily life confronts us with a wide array of social signals that we must perceive and interpret to provide a coherent understanding of the social environment. One of the most important sources of social signals is the face. We use facial cues to develop and maintain successful relationships, and to support social contact that is important for happiness and health. Failures to develop normal social face perception abilities (and reductions in social perception) have been linked with negative psychosocial consequences including social impairment and loneliness^1–4^. With this in mind, the investigation of potential methods to aid social perception is important.

One approach to aid performance that has gained traction in recent years is the use of transcranial electrical stimulation (tES). TES is a noninvasive technique for brain stimulation that can be used to increase or decrease brain activity under a targeted brain region. For example, in one form of tES known as high frequency transcranial random noise stimulation (tRNS) an alternating current ranging randomly between 100–640 Hz is administered between electrodes placed on the scalp, leading to an increase in neural excitability under each scalp electrode^5^. These techniques have commonly been employed with a view to improving perceptual and cognitive performance of participants on a given task (e.g. refs 6–8). Recent work has demonstrated the utility of tES to aid social perception [e.g. refs 9–15]. One example is facial emotion perception. Facial emotion perception plays a key role in interpersonal communication and is a precursor for a variety of socio-cognitive abilities (e.g. empathy). Prior work has suggested that some forms of non-invasive brain stimulation can be useful to aid emotion perception skills^10–15^, but whether tRNS can or cannot aid emotion perception abilities has rarely been addressed in the literature.

In the current investigation we conducted two experiments that sought to determine the efficacy of high frequency tRNS targeted at the inferior frontal cortex (IFC) as a tool to modulate facial emotion perception. The IFC was targeted based on meta-analyses of neuroimaging work pointing to the involvement of bilateral IFC activity during facial emotion perception^16, 17^. Prior work using non-invasive brain stimulation has also shown that modulating neural activity in IFC can influence facial emotion processing: repetitive transcranial magnetic stimulation targeted at the IFC can result in disruption of emotion recognition [e.g. ref. 18], while high frequency tRNS to IFC has been shown to enhance anger perception abilities in older adult participants^15^. These findings highlight the importance of the IFC in emotion processing, but key questions remain. For instance, no studies have assessed the efficacy of using high frequency tRNS targeted at IFC on young adults emotion processing abilities, examined a range of facial emotions, or assessed task and anatomical specificity of effects; we sought to address this for the first time by conducting two experiments.

In Experiment 1, we employed a between participants design comparing two groups of young adult participants on facial identity and facial emotion perception following active or sham high frequency tRNS targeted at the IFC. This enabled us to determine whether high frequency tRNS targeted at the IFC modulated social perception abilities, and if any effects were task specific to facial emotion or facial identity. In Experiment 2, we employed a mixed design comparing facial identity and facial emotion recognition (for happiness, anger, disgust, fear, surprise, and sadness) before and after active high frequency tRNS targeted at the IFC or active high frequency tRNS targeted at area V5/MT (active control site). This enabled us to determine the contribution of individual differences in baseline social perception performance to performance change following tRNS targeted at the IFC, and if any effects were specific to IFC stimulation versus control site stimulation.

## Experiment 1: The role of the IFC in facial emotion and facial identity perception

### Experiment 1 Materials and Methods

#### Participants

All participants were healthy volunteers, without any known developmental or neurological disorders and no contraindications to tRNS. They were naive with respect to the experimental hypothesis and remained unaware of what type of stimulation they received until the end of the experiment. A total of thirty-four participants took part in the study (16 male, 18 female; Mean Age = 23.5 years; SD of Age = 3.41 years). These participants were assigned to either active or sham stimulation. Seventeen participants were in the active group (8 male, 9 female; Mean Age = 23 years, SD of Age = 2.65 years). Seventeen participants were in the sham group (8 female, 9 male; Mean Age = 24 years, SD of Age = 2.65 years). All participants were Caucasian.

#### Brain Stimulation Parameters

High frequency tRNS was administered using a NeuroConn DC Plus Stimulator. Two 5 × 5 cm electrodes placed in saline soaked sponges were used. Stimulation was administered at 1 mA for 20 minutes, with a 15 second fade-in and fade-out time. An identical setup was used for the sham group, but active stimulation was only administered for 15 seconds (plus fade-in and fade-out). This evokes the sensation of being stimulated, but does not lead to a neurophysiological change that can influence performance^19^. The sites of stimulation were identified using the electroencephalography 10–20 system, with electrodes placed over the F7/F8 scalp electrode sites.

#### Materials and Procedure

All participants were provided with written information about the study and a description of the tRNS procedure. The associated safety/risk warnings were explained, and participants were asked to sign an informed consent form. The experiment received full ethical approval by the local ethics committee (Goldsmiths Research Ethics Committee). All methods were conducted in accordance with this and safety guidelines for the use of human participants in non-invasive brain stimulation studies. This was the case for both experiments (i.e. Experiments 1 and 2).

After providing informed consent participants were administered active or sham high frequency tRNS targeted at the IFC. Following stimulation the electrodes were removed, and participants were asked to complete tests of facial emotion and facial identity perception abilities. To ensure comparability between tests we used multiple versions of the Cambridge Face Perception Test (CFPT). The CFPT format can be used to assess facial identity or facial emotion perception under similar conditions. For identity perception, we used the CFPT-Identity (previously called CFPT^20^). In this task participants are shown a target face placed in the top-centre of the screen, with six identity morphs placed in a horizontal row beneath. The six morphs are blends of the target face with different distractor faces (morphs contain either 88%, 76%, 64%, 52%, 40%, or 28% of the target face). Participants are asked to re-order the six morphs from most similar to least similar to the target face. There were two practice trials and sixteen experimental trials in the task, with eight trials displaying upright pictures, and the other eight displaying inverted pictures. To test emotion perception we used two versions of the CFPT that test happiness (CFPT-Happy^21^) and anger (CFPT-Anger^10^) perception skills. In the CFPT-Happy and CFPT-Angry participants are presented with a horizontal row of six faces that are morphed between the target expression and a neutral expression in varying proportions (for CFPT-Happy these proportions are 0%, 3%, 6%, 9%, 12% and 15%; for CFPT-Angry these proportions are 0%, 8%, 16%, 24%, 32% and 40%; note the different proportion morphs are used to avoid ceiling effects on CFPT-Happy). Participants were required to order the faces from the most to the least intense expression of the given emotion. There were ten happy trials and ten anger trials preceded by two practice trials for each emotion type. For all tests (i.e. CFPT-Identity, CFPT-Happy, CFPT-Angry), participants had one minute to complete each trial.

Performance on each task was measured by an error score, which was calculated by summing the deviations from the correct position for each face. One error reflected each position that a face must be moved to be in the correct location. Error scores were summed to determine the total number of errors and this was converted into percentage of correct responses. This approach is consistent with prior work using CFPT measures^9, 10, 15, 21^. Chance performance is 36%^21^.

### Experiment 1 Results and Discussion

**Figure 1:**
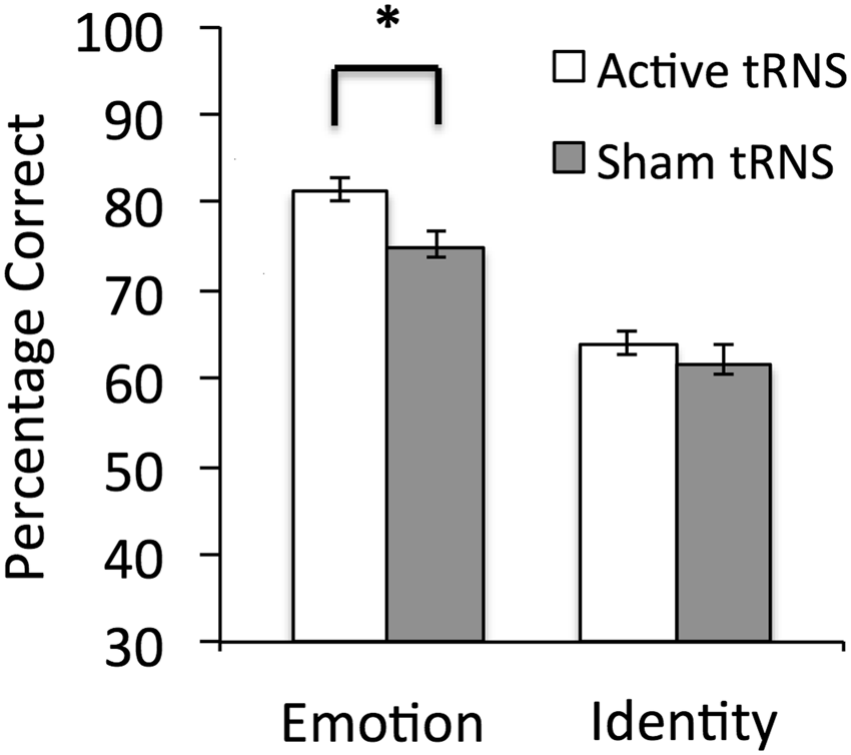
Participants receiving IFC stimulation showed better performance relative to sham on emotion, but not identity perception.

To examine whether performance differed between the active high frequency tRNS and sham participants when completing the facial emotion and facial identity tasks, planned paired comparisons were conducted. These revealed that the active tRNS group outperformed the sham group in emotion perception [t(32) = 2.31, *p* = 0.028, *d* = 0.82], but that the two groups did not differ in facial identity perception [t(32) = 0.793, *p* = 0.434, *d* = 0.28] (Fig. 1).

While these findings suggest that high frequency tRNS targeted at IFC is associated with better emotion perception there are some caveats that need to be considered. For example, prior work suggests that a number of inter-individual variability factors can influence the effects of non-invasive brain stimulation (for review see ref. 22). One important factor is baseline (i.e. no active stimulation) performance level of the participant. This is important in the context of the current results for at least three reasons: 1) although the active and sham tRNS groups differ in emotion performance we cannot be sure that they do not differ in baseline emotion perception abilities, 2) performance was not equivalent between tasks, and 3) prior work has shown that the effects of brain stimulation on behaviour can vary according to baseline performance (e.g. refs 22–27). With this in mind we sought to run a second experiment examining the effects of active high frequency tRNS targeted at the IFC and active high frequency tRNS targeted at MT/V5 on emotion and identity processing abilities before and after stimulation. The inclusion of MT/V5 stimulation as a control brain region was employed to address another shortcoming of Experiment 1 and prior work^15^; namely the inability to make inferences about the anatomical specificity of the active tRNS effects due to the lack of an alternative brain region being stimulated. Collectively this enabled the ability to assess the efficacy of high frequency tRNS targeted at the IFC to modulate social perception abilities (i.e. to replicate Experiment 1); to determine whether these effects were task and anatomically specific; and to identify how individual variability in baseline performance related to performance change following high frequency tRNS.

## Experiment 2: How does individual variability relate to the size of stimulation effects on emotion discrimination following tRNS to IFC and are these effects site specific?

### Experiment 2 Materials and Methods

#### Participants

As with Experiment 1, all participants were healthy volunteers, without any known developmental or neurological disorders and no contraindications to tRNS. They were naive with respect to the experimental hypothesis and remained unaware of what type of stimulation they received until the end of the experiment. No participants from Experiment 1 took part in Experiment 2. Thirty-six right-handed participants took part (19 female, 17 male; Mean Age = 26.34, SD Age = 8.01 years). Participants were randomly assigned to either the IFC (*n* = 18, 10 female, 8 male; Mean Age = 26.1 years, SD Age = 8.3 years) or V5/MT (*n* = 19, 9 female, 10 male; Mean Age = 26.6 years, SD Age = 8 years) stimulation condition. The groups did not differ significantly in age (*t*(33) = −0.201, *p* = 0.842), gender (*x* ^2^ = 0.032, *p* = 0.858) or ethnicity (14 Caucasian, 4 Non-Caucasian in IFC Group; 16 Caucasian, 3 Non-Caucasian in V5/MT; *x* ^2^ = 0.249, *p* = 0.618).

#### Brain Stimulation Parameters

Participants either received active high frequency tRNS to IFC or V5/MT. The location of IFC was identified in the same manner as Experiment 1 using the 10/20 EEG system. V5/MT was also identified using the 10/20 EEG system, with electrodes placed over PO7/PO8 (for similar electrode placement see ref. 28). Ten minutes of stimulation at an intensity of 1 mA using a Neuroconn DC Plus stimulator was given. A shorter duration of stimulation was used to Experiment 1 since the task durations were reduced. Prior work has shown that 10 minutes of active tRNS can lead to increases in cortical excitability for up to 60 minutes following stimulation^5^, which sufficiently covers duration of tasks used in the current experiment. All other stimulation protocol was identical to Experiments 1 (see section 2.1).

#### Materials and Procedure

Participants completed two same-different judgment tasks (from ref. 29). In one task, participants were required to press one of two keys to indicate whether two sequentially presented faces were expressing the same or different emotion. In another participants were required to press one of two keys to indicate whether two sequentially presented faces were the same or different identity. Participants were asked to respond as quickly and accurately as possible. Faces were presented sequentially and appeared on screen for 250ms separated by a fixation cross appearing for 100 ms. Each participant completed 2 blocks (36 trials per block, 72 trials in total) of both the emotion and identity tasks (order counterbalanced) prior to, and following brain stimulation. The stimuli consisted of cropped, grey-scale images of 6 female models displaying one of six emotions (happy, sad, surprise, fear, disgust and anger). The same images were used for both the identity and emotion tasks.

### Experiment 2 Results and Discussion

Performance on each test was assessed using Inverse Efficiency Scores (IES). These were calculated for each session (prior to and following, brain stimulation) and task (emotion and identity) by dividing reaction times by accuracy^30^. IES scores following brain stimulation were then subtracted from IES scores prior to brain stimulation to create a difference score.

Two participants were removed for outlier data (IES difference scores greater than 3 SD from the mean). Therefore data from the remaining 34 participants was analysed (IFC group, *n* = 17, 10 female, Mean Age = 25.3 years SD Age = 7.8 years; V5/MT group, *n* = 18, 9 female, Mean Age = 25.7 years, SD Age = 7.1 years).

**Figure 2:**
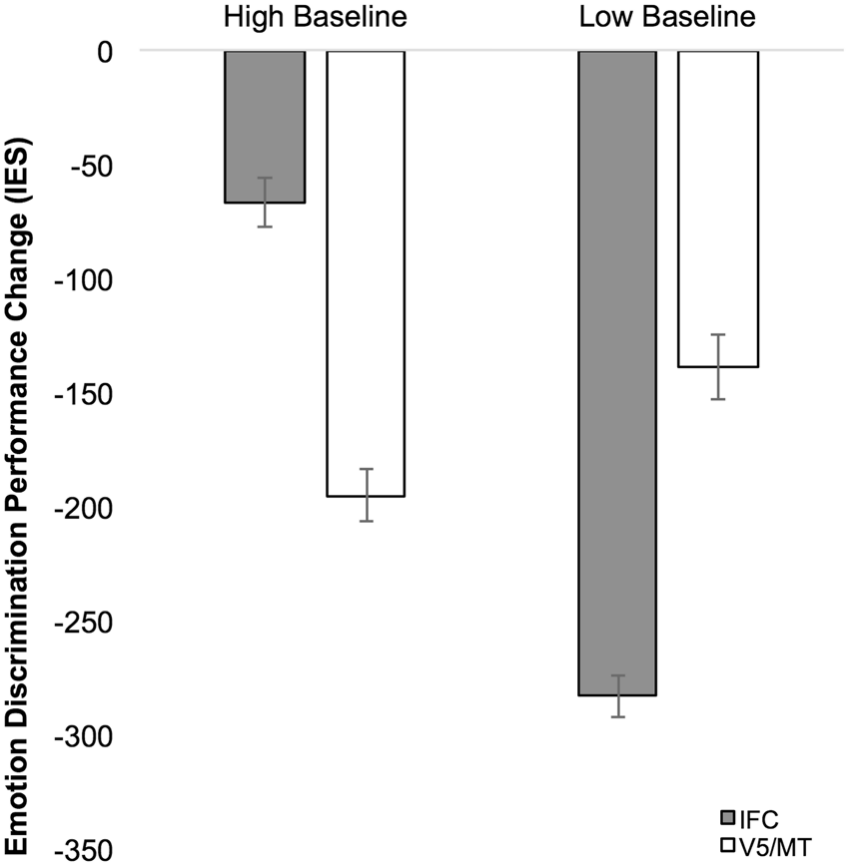
Baseline performance by stimulation site interaction reflecting greatest improvement in low baseline performers on emotion discrimination following IFC stimulation, but not V5/MT.

In order to determine whether baseline levels of performance influenced stimulation effects we conducted a median split using pre-test IES for each of the two stimulation groups. This split between high and low performance was conducted separately for emotion and identity discrimination tasks. Separate 2 (stimulation site [IFC, V5/MT]) × 2 (baseline performance [high, low]) ANOVAs were then conducted to examine the relationship between pre-test performance and performance change on each task. For emotion discrimination, no significant main effects were observed for stimulation site [F(1,30) = 0.026, *p* = 0.874, η_p_^2^ = 0.001] or baseline performance [F(1,30) = 2.547, *p* = 0.121, η_p_^2^ = 0.078]. However, we did observe a significant interaction between stimulation site and baseline performance [F(1,30) = 7.398, *p* = 0.011, η_p_^2^ = 0.198]. Performance improvement was significantly greater for lower, compared to higher, baseline performers following IFC stimulation [*t*(15) = 4.560, *p* < 0.001, *d* = 2.19] (Fig. 2). This effect was not observed for V5 stimulation [*t*(15) = −0.638, *p* = 0.533, *d* = 0.31]. Thus, lower performers showed the greatest gains in performance following active stimulation of IFC in a site-specific manner. This pattern was not observed for identity discrimination performance (see Supplemental Results).

**Figure 3:**
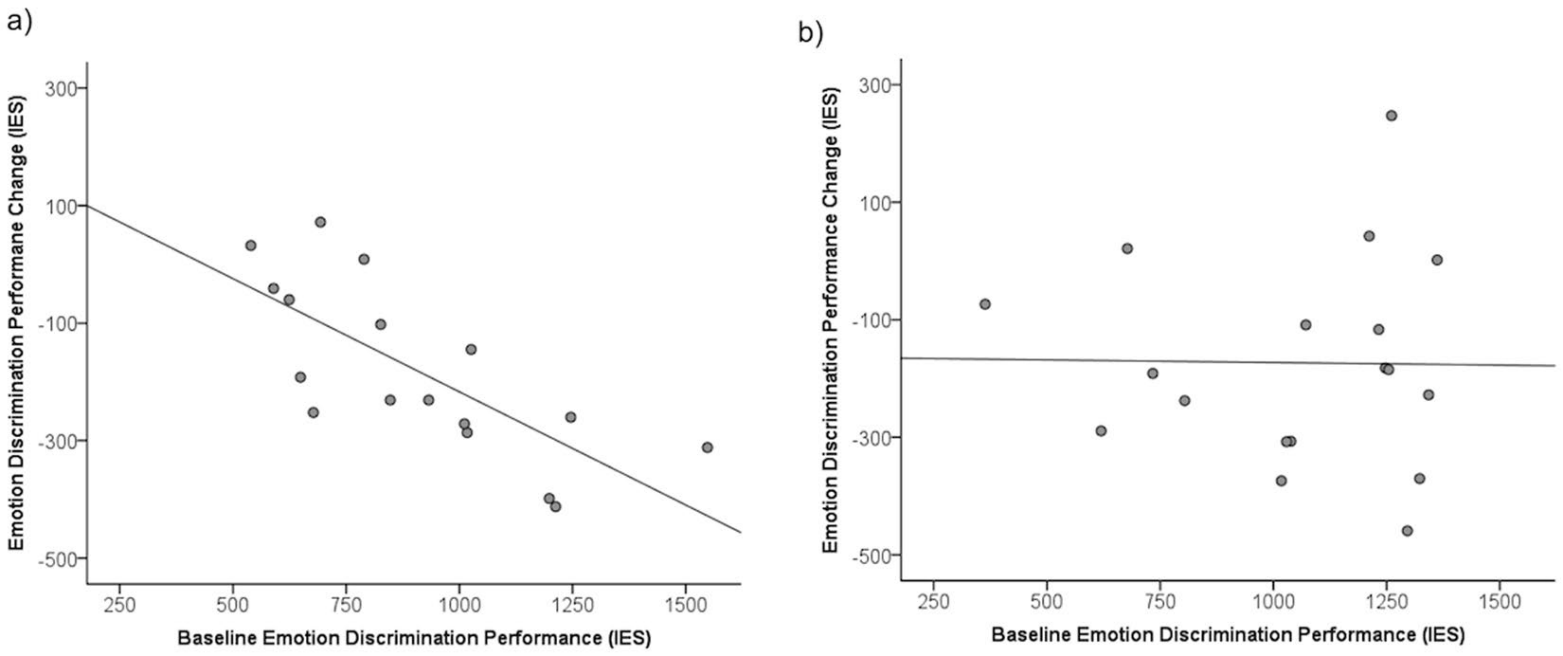
(**a**) The relationship between baseline emotion discrimination performance and performance change following IFC stimulation (more negative scores on the y axis indicate greater improvement). Lower baseline performers show the greatest improvement following stimulation to IFC. (**b**) The relationship between baseline emotion discrimination performance and performance change following V5/MT stimulation (more negative scores on the y axis indicate greater improvement). No significant relationship observed.

To further delineate the relationship between baseline performance and performance change following stimulation to IFC on emotion discrimination, a linear regression was run. Baseline IES scores were entered as the predictor and performance change (difference score between baseline performance and performance stimulation) was entered as the dependent variable. Baseline performance predicted performance change following stimulation to IFC with low performers showing the greatest improvement in performance following stimulation [F(1,15) = 17.82, *p* < 0.001, Adjusted R^2^ = 0.51; baseline performance – *b* = −0.74, *t* = −0.42, *p* < 0.001] (Fig. 3a). Baseline performance also remained a significant predictor when controlling for age, gender and ethnicity [baseline performance - *b* = −0.88, *t* = −3.37, *p* = 0.006; for other variables see Supplemental Table 3]. This pattern was not found following stimulation to area V5/MT: baseline emotion discrimination performance alone [F(1,16) = 0.003, *p* = 0.954, Adjusted R^2^ = −0.06; baseline performance – *b* = −0.02, *t* = −0.06, *p* = 0.954], baseline performance plus age, gender and ethnicity [baseline performance - *b* = 0.009, *t* = 0.03, *p* = 0.977; for other variables see Supplemental Table 3] (Fig. 3b).

These findings provide evidence of a relationship between baseline ability and degree of performance change in emotion discrimination following high frequency tRNS targeted at IFC, but not V5/MT. The results are consistent with Experiment 1 by showing that tRNS targeted at IFC can modulate emotion discrimination abilities, but constrain them by demonstrating that individual variability in baseline emotion discrimination can moderate degree of performance change following tRNS targeted at IFC.

## General Discussion

Building on prior neuroimaging and brain stimulation work suggesting a relationship between neural activity in IFC and facial emotion perception this study sought to examine whether high frequency tRNS targeted at the IFC could result in modulation of emotion perception skills. In Experiment 1, we observed that participants receiving high frequency tRNS to the IFC outperformed those receiving sham stimulation on an emotion, but not an identity perception, task. To investigate this effect further and to determine if individual differences in baseline performance interact with degree of change following stimulation to IFC, we conducted an additional experiment in a different group of participants. Experiment 2 showed that degree of performance change following IFC stimulation was related to baseline emotion discrimination abilities with lower baseline performers showing a significantly greater improvement following stimulation to IFC than high performers. This pattern was not found following active stimulation of a visual control brain region (area V5/MT). It was also not found for identity discrimination. Collectively the experiments indicate that high frequency tRNS targeted at the IFC can modulate emotion perception abilities, but that there are performance moderators of this effect.

The results have a number of implications. Firstly, the findings add to the growing literature showing the potential utility of non-invasive brain stimulation to modulate social processing skills (e.g. refs 9–15). In particular, these findings show the utility of using high frequency tRNS targeted at IFC to modulate emotion processing in younger adults. They highlight the importance of individual differences in the efficacy of outcome following non-invasive brain stimulation, particularly baseline ability. This is in line with recent accounts that highlight the important interplay between a variety of individual differences and transcranial electrical stimulation outcome (e.g. refs 22–27) and studies showing the importance of baseline performance on degree of change following transcranial electrical stimulation (e.g. refs 22–24).

The results also speak to the relationship between IFC activity and emotion perception. Previous research has demonstrated IFC activity during emotion processing tasks^15–18^. This activity is reduced in those with atypical emotion processing abilities^31–33^. This research is consistent with our findings that modulating IFC activity (in this case, through application of tRNS) is associated with better emotion processing performance. Particularly, low performing participants showed the greatest improvement in emotion processing following IFC stimulation. However, high performers showed little change or a minimal decrease in task performance. Delineating the reasons why baseline ability may differentially influence stimulation outcome is an important avenue for future work. It may reflect a number of factors: i) high-performers recruit differential brain networks or rely less on the IFC to support emotion perception, ii) high-performers may benefit less from tRNS as their activated networks are already performing at an optimal level, iii) the importance of IFC activity may vary according to task difficulty.

It is also necessary to consider the possibility that the relationship between baseline performance and performance change following stimulation may reflect regression to the mean^34, 35^. The current findings are unlikely to be explained by this for two reasons. Firstly, low baseline performers show the greatest performance change following tRNS. For regression to the mean to be likely, extreme high performers would be expected to also show a decrease in task performance following stimulation similar in magnitude to the increase observed for low baseline performers - this was not observed. Secondly, while a relationship is observed between baseline emotion processing performance and performance change following tRNS to IFC, a similar trend is not observed for control site stimulation. If the results observed here were reflective of regression to the mean, a similar relationship would be expected regardless of site of stimulation.

A recent study reported that high frequency tRNS targeted at IFC can improve anger perception in older adults, but this is moderated by baseline ability and gender^15^. The findings from that work and our own are largely consistent – namely increased emotion perception following tRNS targeted at the IFC that is influenced by baseline ability. There are, however, some differences to be noted. Firstly, in our current work we included task and site controls, thereby enabling us to making stronger claims regarding the anatomical and task specific nature of the IFC in emotion perception. Secondly, we test a wider range of emotions and observe overall task improvement (note, we do not break down by emotion type due to the low number of trials per type). In future work it will be interesting to directly compare how tRNS to IFC influences the perception of different emotion types in younger and older adults using the same study design.

In addition to tRNS studies, previous research has demonstrated that anodal tDCS to the left dorsolateral prefrontal cortex (DLPFC) enhances emotion recognition in a group of controls and individuals with depression^12^. In that study, there was also evidence of modulated working memory abilities, which may have contributed to the emotion processing changes. In a similar context, another group of authors report that anodal tDCS targeted at right oribtofrontal cortex (OFC) resulted in better performance on an emotion-labelling task in female typical adults^13^. While our stimulation was targeted at IFC we cannot rule out the possibility of activation of nearby brain regions. In this regard, our data could be considered to have some degree of consistency with this prior work, and to extend it by showing that emotion processing was modulated following frontal stimulation even when working memory and vocabulary demands were low (in Experiment 1). There are also some other important differences to note between our study and prior work including a) the use of a different form of stimulation that may act on different mechanisms^36^, b) differences in sample, and c) differences between bilateral and unilateral stimulation.

Another relevant theoretical issue that will be an interesting avenue for further study is why exogenous manipulations of IFC changed emotion, but not facial identity task performance. One possibility is that this is contingent on the degree to which successful performance on each task type (i.e. emotion/identity) is related to the use of implied movement cues. Although linked to emotion perception, there is also evidence that the IFC can alter the ability to recognise others’ emotionally neutral actions^37–39^. It is possible that the emotion recognition tasks used rely more on the processing of implied facial movements that characterise specific emotional expressions, whereas the facial identity tasks are more reliant on the processing of facial morphological features rather than facial movements. In this regard, it could be argued that tRNS over IFC acted on the processing of implied action, rather than being specific to the processing of emotions. This will be an interesting question to address with future studies.

In summary, the data presented demonstrate that a single-session of tRNS to the IFC can modulate emotion perception skills relative to sham and active control site stimulation in younger adults, but that this effect is influenced by baseline performance. This is consistent with brain imaging data highlighting the role of IFC in emotion perception and prior tRNS work in older adults. It is also in line with opinions in the field of brain stimulation research suggesting that individual differences play an important role in moderating the effects of stimulation.

## Electronic supplementary material

Supplementary Results
