# Supplementary material for: Emotion perception improvement following high frequency transcranial random noise stimulation of the inferior frontal cortex
Source: Sci Rep. 2017 Sep 12;7:11278. doi: 10.1038/s41598-017-11578-2 (PMC5595798; doi:10.1038/s41598-017-11578-2)
Supplement: Supplementary file 1 — Supplementary Results [file 41598_2017_11578_MOESM1_ESM.pdf]

Supplementary Results for ‘Emotion perception improvement following high frequency transcranial random noise stimulation of the inferior frontal cortex’

Tegan Penton[1], Laura Dixon[2], Lauren Jayne Evans[2], and Michael J Banissy[2*]

1. MRC Social, Genetic and Developmental Psychiatry Centre, Institute of Psychiatry, Psychology and Neuroscience, King’s College London, Denmark Hill, London, SE5 8AF

2. Department of Psychology, Goldsmiths, University of London, New Cross, London, SE14 6NW

*Correspondence should be addressed to:

Dr Michael Banissy

Department of Psychology

Goldsmiths

University of London

New Cross

London

SE14 6NW

m.banissy@gold.ac.uk

Experiment 2 – Identity Discrimination Results

To examine whether baseline levels of identity discrimination performance may interact with stimulation effects we conducted a 2 (stimulation site [IFC stimulation, V5/MT stimulation]) x 2(baseline performance [high, low]) ANOVA to examine the relationship between pre-test performance and performance change on the identity discrimination task. A significant main effect was observed for baseline performance [$F(1,29) = 5.07$, $p = .032$, $\eta_p^2 = .149$] indicative of lower baseline performers showing the greatest improvement in identity discrimination regardless of stimulation site. There was no significant main effect of stimulation site [$F(1,29) = 0.12$, $p = .731$, $\eta_p^2 = .004$] and no significant site x baseline performance interaction [$F(1,29) = 0.15$, $p = .705$, $\eta_p^2 = .005$].

Supplemental Table 1. Descriptive statistics for accuracy (% correct), reaction time (RT in milliseconds), and inverse efficiency (IES in milliseconds) in the emotion discrimination task for each performance group (high/low baseline performance – each identified separately per dependent variable) across stimulation conditions.

Task & DV	Performance Group	Stimulation Site	N	Baseline (SD)	Post-Stimulation (SD)	Difference (SD)
Emotion (Accuracy)	**High Performers**	IFC	10	82.03 (5.14)	83.96 (7.02)	1.92 (6.41)
		V5	9	81.99 (2.53)	79.9 (7.31)	-2.09 (5.94)
	**Low Performers**	IFC	7	73.59 (2.74)	76.8 (4.67)	3.21 (5.34)
		V5	9	71.41 (6.78)	71.39 (7.1)	-0.03 (6.45)
	**Overall**	IFC	17	78.56 (6)	81.01 (7.01)	2.45 (5.82)
		V5	18	76.71 (7.36)	75.65 (8.25)	-1.06 (6.11)
Emotion (RT)	**High Performers**	IFC	11	554.09 (91.46)	480.55 (106.94)	-73.55 (63.75)
		V5	7	542.07 (123.14)	443.64 (157.78)	-98.43 (94.63)
	**Low Performers**	IFC	7	904.79 (102.74)	708.86 (95.76)	-195.93 (48.63)
		V5	11	953.32 (102)	786.23 (176.21)	-167.09 (154.05)
	**Overall**	IFC	18	690.47 (198.98)	569.33 (151.92)	-121.14 (83.63)
		V5	18	793.39 (232.44)	653 (237.87)	-140.39 (135.3)
Emotion (IES)	**High Performers**	IFC	8	673.76 (96.46)	606.84 (139.94)	-66.92 (111.32)
		V5	8	789.6 (243.56)	594.56 (217.49)	-195.15 (133.28)
	**Low Performers**	IFC	9	1115.5 (210.44)	832.38 (184.99)	-283.11 (83.72)
		V5	9	1281.07 (52.12)	1142.22 (205.37)	-138.85 (215.2)
	**Overall**	IFC	17	907.62 (279.04)	726.24 (197.85)	-181.37 (145.94)
		V5	18	1049.16 (293.06)	875.96 (339.67)	-173.2 (176.09)

Supplemental Table 2. Descriptive statistics for accuracy (% correct), reaction time (RT in milliseconds), and inverse efficiency (IES in milliseconds) in the identity discrimination task for each performance group (high/low baseline performance – each identified separately per dependent variable) across stimulation conditions.

Task & DV	Performance Group	Stimulation Site	N	Baseline (SD)	Post-Stimulation (SD)	Difference (SD)
Identity (Accuracy)	**High Performers**	IFC	8	87.57 (4.17)	89.76 (5.24)	2.19 (6.6)
		V5	10	89.12 (3.12)	91.5 (4.77)	2.37 (3.25)
	**Low Performers**	IFC	9	74 (8.28)	81.15 (4.8)	7.15 (10.77)
		V5	8	70.93 (11.32)	76.74 (13.07)	5.8 (6.49)
	**Overall**	IFC	17	80.39 (9.52)	85.2 (6.57)	4.81 (9.14)
		V5	18	81.04 (12.02)	84.94 (11.81)	3.9 (5.1)
Identity (RT)	**High Performers**	IFC	10	457.2 (80.95)	411.5 (75.24)	-45.7 (38.65)
		V5	8	474.88 (89.71)	395.94 (109.1)	-78.94 (91.67)
	**Low Performers**	IFC	8	703.13 (63.92)	598.94 (87.55)	-104.19 (96.12)
		V5	10	759.95 (109.39)	651.75 (122.22)	-108.2 (59.06)
	**Overall**	IFC	18	566.5 (144.79)	494.81 (123.85)	-71.69 (74.09)
		V5	18	633.25 (175.77)	538.06 (172.97)	-95.19 (74.37)
Identity (IES)	**High Performers**	IFC	9	896.68 (194.75)	698.43 (114.39)	-198.26 (201.68)
		V5	9	1027.46 (230.16)	827.39 (195.14)	-200.06 (104.65)
	**Low Performers**	IFC	8	556.35 (115.68)	480.25 (105.34)	-76.1 (62.58)
		V5	8	569.31 (94.46)	459.37 (126.74)	-109.94 (102.82)
	**Overall**	IFC	17	736.53 (235.54)	595.76 (154.91)	-140.77 (161.25)
		V5	18	827.19 (291.76)	648.19 (242.75)	-179 (140.48)

Supplemental Table 3. Values of additional predictor variables entered into emotion discrimination regressions.

**Stimulation**	**Predictor**	***t***	***b***	***p***
**IFC**	Age	.15	.03	.88
	Gender	-1.04	-.21	.32
	Race	.28	.07	.79
**V5**	Age	.45	.15	.67
	Gender	.82	.29	.43
	Race	-.37	-.11	.72
